# Supplementary material for: Are carbon emissions trading and green financial instruments synergistic? -Comprehensive quantitative research based on content analysis
Source: PLoS One. 2024 Mar 7;19(3):e0298601. doi: 10.1371/journal.pone.0298601 (PMC10919601; doi:10.1371/journal.pone.0298601)
Supplement: S1 Data — (ZIP) [file pone.0298601.s001.zip › Data/地级市Z_F工作报告.zip!/╣π╬≈/║╪╓▌╩╨/└┤╫╘╣1⁄2╓┌║┼í╛┬φ┐╦╩2╛▌═°í┐_2006.docx]

各位代表：

　　现在，我代表市人民政府向大会作关于贺州市国民经济和社会发展第十一个五年规划纲要的报告，请各位代表连同《纲要（草案）》一并审议，并请各位政协委员和其他同志提出意见。

“十五”时期工作回顾

　　“十五”时期，是我市发展史上不平凡的时期，也是我市经济社会发展速度最快、群众得到实惠最多的时期。五年来，我们在自治区党委、自治区人民政府和市委的领导下，在市人大及其常委会的监督和市政协的支持下，团结和带领全市人民，在完成撤地设市任务的同时，坚定不移地推进“三化两业”，全面完成了国民经济和社会发展第十个五年计划，全市经济社会持续快速健康发展，人民生活不断改善，改革、发展、稳定各个方面都取得重大成就，为“十一五”时期实现更快更好发展奠定了坚实基础。

　　综合经济实力迈上新台阶。初步统计，全市生产总值由2000年的94.5亿元增加到2005年的164.3亿元，年均增长10.4％，人均生产总值由2000年的705美元提高到1060美元。财政收入由2000年的5.69亿元增加到2005年的11.22亿元，年均增长17.7%。三次产业结构由2000年的41.9：26.7：31.4调整为32.0：43.4：24.6，经济结构由以农业为主向工业主导格局转变。

　　工业成为经济增长的主要力量。初步统计，“十五”时期工业增加值年均增长14.5％，规模以上工业企业盈亏相抵后实现利润总额年均增长24.8%。工业对经济增长的主要作用日趋明显，工业增加值对经济增长的贡献率年均达到32%，优势产业和强优企业发展壮大。桂东电子中高压电子铝箔、灵峰药业GMP认证等重点技改项目以及真龙彩印烟用商标印刷、金源稀土高纯稀土功能材料等新项目建成投产，大锰银鹤无汞碱性电池生产线、英格瓷矿业重钙超细粉、嘉和木业中纤板等项目开工建设。　　旺高工业区、信都工业区和平桂西湾工业基地建设取得显著成效，进入旺高工业区的企业达21家。

　　城乡建设成效显著。城乡规划、建设和管理水平提高，完成了贺州市城镇体系规划修编和一批控制性详规、专项规划编制。汇豪国际城、远东国际城、市客运中心、体育中心等项目开工建设，建设路和贺州大道改造、管道燃气、贺州学院新校区等项目加紧推进，龟石供水一期工程、南环路一级水泥路改建等一批城市道路、供电、供排水、通讯等基础设施项目建成投入使用，城市建成区面积由“九五”期末的15平方公里扩大到23平方公里。城市绿化、美化、亮化、净化水平提高，市容市貌焕然一新，在全区第五届市容环境综合整治“南珠杯”竞赛届末评比中荣获优秀奖第一名，位居五个新设地级市之首。各县城区和重点镇的建设也取得新成绩。城镇化进程进一步加快，城镇化率由“九　五”期末的21.3%提高到30%。

　　农业和农村经济全面发展。农业综合生产能力不断提高，农林牧渔业总产值年均增长4.4%。特色农业、品牌农业加快发展，无公害农产品行动计划和农产品标准化生产取得新成绩，无公害蔬菜、脐橙、烟叶、马蹄、茶叶、梅李等十大优势农产品基地进一步扩大，桑蚕、食用菌等新兴农产品基地快速发展。畜牧水产业良种化、规模化、标准化建设取得新进展。生态林业建设进一步加强，速丰林、松脂林基地建设稳步推进。农业企业化步伐加快，新增规模农业龙头企业265家。农田水利、人畜饮水、电网、乡村道路等农村基础设施建设得到加强，农村生产生活条件不断改善，率先在全国建成中国水电电气化市。

　　商贸旅游服务业发展水平提高。主要旅游景区景点基础设施不断完善，宣传促销力度加大，服务水平不断提升。2005年接待国内外游客275.3万人次，旅游总收入12.9亿元，分别比“九五”期末增长2.9倍和8.6倍，年均增长31.5%和57.1%。服务业基础设施明显改善，盛隆百货、新时代购物广场等大型商场竣工开业，桂湘粤农贸市场、金旗市场、鼎富装饰广场、商业步行街等项目加快建设。引进了南宁百货、肯德基等商业企业。服务业结构进一步优化，房地产业发展势头强劲，商贸流通业活跃繁荣，金融保险业加快发展。

　　基础设施和重大项目建设取得突破。百项工程大会战和交通基础设施建设大会战深入实施，固定资产投资强劲增长。“十五”时期全社会固定资产投资年均增长51.2%，累计完成174.7亿元,超过前九个五年计划完成的投资总和，投资对经济增长的贡献率年均达到52%。洛湛铁路、桂梧高速公路、广贺高速公路贺州段和国道323线莲塘至鹰阳关二级公路改造、贺江复航工程等“南北贯通，五龙出海”大通道项目开工建设，上程电站、下福电站、金牛坪电站等水电项目加快推进，桂东火电厂、永州至贺州高速公路等重大项目前期工作取得积极进展。

　　改革开放取得明显成效。国有企业改革取得实质性进展，顺利完成平桂、西湾矿务局和钟山、富川烟厂的关闭破产以及平桂医疗系统改制和市二机厂等企业改制。农村税费改革步伐加快，取消烟叶以外的特产税和降低农业税税率，提前一年免征农业税，直接减轻农民负担9058万元。非公有制经济进一步发展。做好了两届中国—东盟博览会的参展招商工作，成功举办两届农产品展示会、亚细安（东盟）客属第六届恳亲会暨贺州市经贸项目洽谈会等招商推介活动，加强与广东佛山、浙江衢州等地以及柳州铁路局的交流合作，招商引资成效显著。五年累计引进内资项目2039个，利用外来资金118亿元；引进外资项目82个，实际利用外资6400万美元，分别是“九五”时期的2倍和1.3倍。外贸出口额累计完成41706万美元，年均增长11.8%。

　　城乡人民生活进一步改善。初步统计，城镇居民人均可支配收入年均增长12.2%，农民人均纯收入年均增长7.3%。市场消费持续旺盛，社会消费品零售总额年均增长10.8%。累计建成五保村721个，安置五保老人14648人，率先在全国推行农村五保老人集中供养。完成农村贫困残疾人危房改造900户。新阶段扶贫开发取得成效，贫困人口减少8.95万人。就业和再就业工作进一步加强，社会保障体系不断完善。五年新增城镇就业5.64万人，1.97万名失业和下岗职工实现再就业，城镇登记失业率控制在3.8%以内。培训农村富余劳动力35.53万人，新增劳务输出11.2万人。

　　各项社会事业全面进步。第二、三轮创新计划取得新成果，科技创新能力进一步提高。“两基”成果得到巩固发展，办学条件不断改善,教育教学质量明显提高。贺州学院申办顺利通过设置评议。加强人才的培养、引进和使用，人才队伍不断壮大。舞台艺术精品创作成果喜人,文化事业和文化产业加快发展。广播电视 “村村通”阶段性目标任务提前超额完成，广播电视覆盖率达到87.6%。新闻出版事业有新的发展,“扫黄打非”工作取得新成效。公共卫生体系进一步完善，疾病预防控制能力得到增强。全民健身活动广泛开展，成功举办了市第一届体育运动会。群众性精神文明创建活动广泛开展。资源合理开发、环境保护和生态建设不断加强。创新并落实计划生育奖励扶助和利益导向机制，低生育水平持续稳定。国防建设和民兵预备役工作继续推进，双拥工作扎实开展，荣获自治区“双拥”模范城称号。“四五”普法取得明显成效。“平安贺州”建设扎实推进，“三无村屯（社区）”创建活动深入开展，重视信访、人民调解和群体性事件的排查和妥善处置，治安、刑事案件明显下降。安全生产工作进一步加强。药品、食品放心工程深入实施，保障了人民群众的健康和安全。依法加强民族、宗教事务管理。妇女、儿童、老年人、残疾人权益得到较好保障。机构编制、档案、地方志工作稳步推进。外事、侨务、对台工作得到加强。技术监督、工商管理、食品药品监督、供销合作、民政、审计、统计、物价、气象、人防、地震、工会等工作取得新成绩。

　　行政效能明显提高。较好地完成了撤地设市后政府职能和管理方式的转变。新一轮政府机构改革稳步实施，行政审批制度改革不断深化，取消和调整行政审批项目，设立政务中心，政务公开和电子政务积极推进，政府公共服务水平进一步提高。行政许可法深入实施，依法行政水平有新提高。科学民主决策机制不断完善，广泛听取和采纳社会各方面的意见和建议，自觉接受人大法律监督和政协民主监督，认真办理人大代表议案、建议和政协委员提案，加强与各民主党派、工商联、人民团体和无党派人士的联系。保持共产党员先进性教育、机关效能建设和创建学习型、创新型领导班子等活动有效开展，公务员队伍建设进一步加强。廉政建设和反腐败斗争深入推进，政风建设取得新成效。

　　“十五”期间取得的巨大成就，使我市进入了基础增强、发展提速的新阶段。特别是2005年，全市经济社会继续保持近年来快速发展的好势头，主要指标增幅再创新高，完成了市一届人大四次会议确定的目标任务，为“十五”计划的全面完成划上了圆满句号。初步统计，2005年全市生产总值164.3亿元，比上年增长14.0％，三次产业分别增长5.2%、23.9%和9.8%。财政收入突破10亿元大关，实现11.22亿元，增长27.9％。工业增加值58.1亿元，增长21.7％。全社会固定资产投资82.96亿元，增长80.4％。社会消费品零售总额34.5亿元，增长13.7%。引进内资项目609个，利用外来资金59.39亿元，增长49.97%；引进外资项目19个，实际利用外资629.83万美元。城镇居民人均可支配收入7527元，增长19.4%；农民人均纯收入2351元，增长12.5%。

　　各位代表，过去的五年，我们不仅面临着机关搬迁和产业结构调整的困难，还面临着连年发生旱涝灾害，煤电油运紧张，土地、资金和重要原材料制约严重等诸多困难。在困难面前，我们迎难而上，奋力拼搏，取得了超出预期的喜人成绩。这是自治区党委、自治区人民政府和市委正确领导的结果，是全市广大干部群众团结一致、共同努力的结果。在此，我代表市人民政府，向全市广大干部群众，向给予我们支持与监督的人大代表、政协委员、各民主党派、工商联和无党派人士、各人民团体、社会各界人士，向大力支持我们工作的中直、区直驻贺单位和人民解放军、武警部队官兵，表示崇高的敬意和衷心的感谢！

　　过去的五年，我们探索和积累了一些加快经济社会发展的宝贵经验：一是坚持落实科学发展观，走符合贺州市情的发展路子。我们认真贯彻中央和自治区的方针政策，从市情出发，提出了“三化两业”的阶段性工作重点，坚持“五业并举，工业优先，夯实基础，做大总量”的经济工作要求，使各项工作都开创了新局面，实现了经济社会的持续快速发展。二是坚持狠抓项目建设，充分发挥投资的拉动作用。我们坚持以项目为载体，实施百项工程大会战、交通基础设施建设大会战，统筹推进一大批覆盖经济和社会各领域的重大项目建设，努力解决项目建设中资金、土地等方面存在的问题，保持了投资对经济增长的强劲拉动，进一步夯实了加快发展的基础。三是坚持创优环境，加快改革开放步伐。我们坚持抓好软硬环境建设，着力消除经济社会发展中的各种障碍，紧紧抓住中国—东盟自由贸易区和泛珠三角经济区构建、西部开发深入实施的机遇，主动融入粤港澳，拓展闽苏浙，挺进东南亚，全面加强国际国内区域合作，招商引资水平不断提高，为经济社会发展注入了强大动力。四是坚持重视和改善民生，努力构建和谐社会。我们把实现好、维护好、发展好人民群众的根本利益作为工作的出发点和落脚点，大力实施并拓展六项“民心工程”，努力为民办实事办好事，使人民群众从改革发展中得到更多实惠。五是坚持大力弘扬高效务实的工作作风，不断提高工作创新力和执行力。我们着力推进政府职能转变，抓好干部队伍建设，强化工作责任制，努力建设服务型政府，使公务员队伍始终保持了奋发有为、昂扬向上的精神状态和团结拼搏、创新务实的工作作风，用心做事，用智慧做事，形成了加快经济社会发展的强大合力。

　　回顾五年来的工作，我们也清醒地认识到，我市经济社会发展中还存在不少困难和问题。主要是：经济总量偏小，财力还比较薄弱；经济结构不够合理，工业化程度不高；城镇基础设施不够完善，城镇化水平偏低；制约发展的瓶颈因素较多，加快发展的基础还不牢固；农民收入水平还不高，就业形势依然严峻；发展环境仍需进一步改善，行政效能有待进一步提高等等。对这些困难和问题，我们将高度重视并采取有效措施努力加以解决。

“十一五”发展目标和任务

　　“十一五”时期是我市经济社会发展承前启后、实现跨越式发展的关键时期。我们面临的形势仍将是机遇与挑战并存、机遇大于挑战、内外环境有利于加快发展。我们一定要认清形势，抢抓机遇，明确“十一五”时期发展的基本思路和任务，积极应对困难和挑战，扎实推进各项工作，实现经济社会更快更好发展。

　　“十一五”时期，我们必须坚持以邓小平理论和“三个代表”重要思想为指导，认真贯彻党的十六大和十六届五中全会以及自治区党委八届六次全会精神，以科学发展观统领经济社会发展全局，继续以财政增长、居民增收为基本目标，以项目建设为载体，以招商引资和发展非公有制经济为突破口，以推进“三化两业”为重点，以提高人民生活水平和促进人的全面发展为根本出发点和归宿，按照“五业并举，工业优先，夯实基础，做大总量”的经济工作要求，加快改革开放步伐，增强自主创新能力，推进经济结构调整，转变经济增长方式，扩大经济总量，增强经济发展后劲，全面发展社会事业，切实改善民生，不断提高人民生活水平，促进和谐社会建设，努力把贺州建设成为经济繁荣、社会进步、环境优美、充满活力的区域性中心城市。

　　根据面临的形势和市委一届五次全会《建议》精神，《纲要（草案）》提出了我市“十一五”期间经济社会发展的目标：

　　──经济总量要上新台阶。在优化结构、提高效益和降低消耗的基础上，实现生产总值年均增长12％左右，2010年人均生产总值比2000年翻一番以上；财政收入年均增长15%左右；全社会固定资产投资年均增长25％左右。

　　──经济结构要上新层次。三次产业结构比例提升为 22.8:52:25.2。工业增加值占生产总值比重达到46.3%，工业主导型经济格局基本形成。非公有制经济占经济总量的比重有较大幅度提高。

　　──统筹城乡发展要有新突破。城市基础设施进一步完善，区域性中心城市的集聚辐射能力显著增强。县城和小城镇加快发展，期末城镇化率达到38％左右。县域经济快速发展，县域单位平均生产总值达70亿元以上。

　　──改革开放要上新水平。经济社会发展的体制和机制性障碍进一步消除，社会主义市场经济体制进一步完善；区域合作与交流进一步扩大，开放型经济达到新水平。

　　──社会事业要有新进步。自主创新能力显著提升，高新技

术产品产值占工业总产值的比重明显提高；九年义务教育得到巩固提高，高等教育在校生达到1万人以上，职业教育招生规模与普通高中大体相当；公共卫生和医疗服务体系不断完善；社会保障体系更加健全，农村社会保障体系初步建立，城镇企业职工基本养老保险、失业保险和城镇职工基本医疗保险覆盖率明显提高。

　　──资源环境状况要有新改善。资源利用率显著提高，万元生产总值能源消耗比“十五”期末明显降低；生态环境进一步改善，森林覆盖率达到70%；人口自然增长率控制在8‰以下。

　　──人民生活水平要有新提高。城镇居民人均可支配收入年均增长10%左右，农民人均纯收入年均增长9%左右。城乡就业扩大，城镇登记失业率控制在4%以内。

　　《纲要（草案）》提出，实现上述目标，要全力实施好六项重点任务：

　　一、大力推进“三化两业”，不断提高发展的速度和质量

(一)以培育发展优势产业集群和强优企业为重点，加快推进工业化。坚持工业优先发展，按照“大改革，大企业，大群体，大产业”的思路，培育发展优势产业集群和强优企业，大力发展电力、矿产、建材、林产、农产品加工、电子、制药等优势产业，引导企业加大技改投入，运用高新技术和先进适用技术改造提升传统产业，打造知名品牌，推动产业优化升级，加大产业配套力度，延长产业链，壮大工业总量。加强石材加工、有色金属加工、粉体材料深加工三大矿业基地建设，培育发展有色金属及稀有金属深加工、石材开采与加工、非金属粉体加工、铁锰综合开发等四大支柱矿业。积极发展替代产业、后续产业和高新技术产业, 构建新的产业发展格局。鼓励和支持重点企业做大做强，着力培植壮大一批具有核心竞争力的骨干企业。进一步完善政策措施，加强协调服务，支持和引导中小企业发展。大力发展园区经济，创新园区管理和利益分配机制，加快工业园区基础设施建设，加大招商引资工作力度，加快项目入园进区步伐，使旺高工业区、信都工业区和平桂西湾工业基地尽快形成各具特色、优势互补的产业群，成为承接东部产业转移的重要基地。同时，加快各县（区）工业基地建设，着重抓好八步贺街、钟山城郊、昭平潮江、富川新湾等工业小区建设，发展壮大县域工业经济。

(二)以贺州城区建设为重点，加快推进城镇化。围绕构建区域性中心城市的目标，培育发展以贺州城区为核心，以县城为骨干，重点镇和其他城镇为基础梯级发展的城镇体系。加快城市基础设施建设，提升经济发展的支撑能力。加快城市路网建设，按照“东西延伸，南北拓展”的要求，重点建设江南新区和城西区，拓展城市规模。建设市物流中心、奥林匹克体育中心、会展中心、太白湖公园、大钟山公园、垃圾处理厂、污水处理厂等项目，进一步完善城市基础设施，实现“五年创新城”目标。优先发展城市公共交通。加强城市人防工程建设。推进城市经营，培育多元化投资主体，广泛吸纳社会资本参与城市建设、经营和管理。建立健全长效管理机制，全面提升城市管理水平。根据功能定位和发展模式，加快县城建设，完善县城基础设施，发展县域特色经济，提高承载能力。加强重点镇建设，突出加强高速公路、铁路沿线重点镇建设，形成新的产业、人口和物流集聚点。

(三)以优势农产品基地建设为重点，加快推进农业企业化。坚持用抓工商业的理念发展农业，积极引进和培育发展一批以农产品加工为重点的农业龙头企业，打造农业企业知名品牌。大力发展品牌农业。在确保粮食安全的前提下，持续推进农业结构调整，重点抓好优势农产品基地建设，培育发展优势农产品产业集群，推动农业生产经营向规模化、标准化、品牌化转变，进一步强化我市作为粤港澳地区“菜篮子”重要基地的优势地位。大力发展瘦肉型猪、优质肉牛、优质家禽和名优水产，提高畜牧水产业占农业总产值的比重。加快发展生态农业，全面实施农产品标准化生产和无公害农产品行动计划，积极开展优质农产品“三品”产地认定、产品认证和申请商标注册及原产地注册，培育和创建一批农产品品牌。加强生态富民家园的规划建设，建设一批生态富民示范村。

(四)以生态旅游和现代物流为重点，巩固提高以商贸旅游业为主的第三产业。围绕创建中国优秀旅游城市的目标，按照“提升品位、形成合力、打造品牌、快速发展”的要求，加快发展旅游业。以主要景区景点基础设施为重点，加强旅游业基础设施建设，努力改善旅游业基础条件。整合开发旅游资源，打造生态旅游品牌，增强旅游产业的核心竞争力。加强区域旅游合作，加快与珠三角、大桂林旅游圈的合作与发展，实现旅游线路对接、资源共享和客源互流。大力发展旅游相关产业，加快星级宾馆、娱乐、休闲、餐饮、购物等设施建设。积极开发具有贺州特色的旅游商品，建设旅游工艺品和特色餐饮一条街，提升旅游产业层次。以建设区域性商贸中心和物流中心为目标，加快发展商贸服务业。编制贺州市商贸流通业发展总体规划和商业网点规划，建设各类专业市场和大型购物设施，抓好酒店、餐饮、娱乐业基础设施建设。引进、扶持和培育商贸龙头企业。运用现代服务技术和经营方式改造提升商贸流通、餐饮等传统服务业，大力发展连锁经营、物流配送、房地产、金融保险、社区服务、信息中介、电子商务等现代服务业。加强信息化建设，提高信息化服务水平。

　　二、改善发展基础条件，统筹城乡协调发展

（一）加强以交通、能源为重点的基础设施建设，提高基础设施水平。以构建区域性交通枢纽城市为目标，深入实施交通基础设施建设大会战，努力构建“南北贯通，五龙出海”交通新格局，形成贯通内外、连接城乡、畅通快捷的交通运输网络。加快洛湛铁路、桂梧高速公路、广贺高速公路贺州段和323国道改造、贺江复航工程建设，积极配合做好广州经贺州至贵阳干线铁路、永州至贺州高速公路项目的前期工作，争取早日动工建设。全面实施路网建设工程，建设连通高速公路的路网连线以及与珠三角、周边市（县）对接的快速通道，改造一批技术等级和路面等级低的县乡道路，力争期末实现县城二级以上公路与高速公路连接，全部乡镇通等级油路或水泥路，全部行政村通等级公路，其中50%以上的行政村通等级油路或水泥路。加快建设贺州港信都、昭平作业区，整治贺江和桂江航道，实现贺江复航。加快市客运中心以及各县（区）和重点乡镇客运站等运输站场建设。大力推进以电力为主的能源建设。建成一批重点水电项目。积极开展桂东火电厂、上程抽水蓄能电站等项目前期工作，争取早日动工建设。加快与南方电网、华中电网对接的电网建设，重点建设八步至南方电网500千伏变电站等输变电工程，建成“西电东送”的重要基地。加强水利设施建设。加快实施贺州及各县城区防洪工程，完成市区防洪排涝一期工程建设，开工建设昭平城区防洪排涝工程，争取富川、钟山县城区重点堤段防洪排涝工程开工建设。加强病险水库的除险加固。建设水库群信息采集和调度系统，加快水利灌溉工程和农田水利设施建设，重点实施龟石、合面狮、龙潭、鸟源四个大中型灌区节水改造工程。重视城市饮水安全，实施贺州水厂、昭平水厂供水扩建工程，完善市区和钟山、富川城区供水管网，建设一批城镇供水和农村饮水安全工程。

（二）积极推进社会主义新农村建设，加快发展农业和农村经济。按照“生产发展、生活宽裕、乡风文明、村容整洁、管理民主”的要求，以增强农业综合生产能力和促进农民增收为核心，以农村基础设施建设为突破口，扎实推进新农村建设。突出抓好农村道路、水利、能源、教育、卫生等基础设施和公共设施建设，不断改善农村生产生活条件。积极推进农村综合配套改革，继续落实各项支农惠农政策，不断增强农业和农村发展活力。建立健全农业科技推广应用、质量监测、质量认证和病虫害防治等农业服务体系。扶持壮大农村经济合作组织，提高农业组织化程度，促进农产品流通。加大农村富余劳动力转移就业培训，大力发展劳务经济，增加农民收入。

（三）发展壮大县域经济，提高整体发展水平。大力发展县域个体、私营等非公有制经济。挖掘资源优势，培育壮大优势产业，形成区域特色经济，促进县域经济发展。扶持发展县域中小企业，引导各类项目和乡村工业向工业基地集中，加快县域工业发展，壮大县域经济主体。切实加强县城和重点镇建设，提高承载能力，扩大县城人口规模，鼓励和引导外出务工人员到县城居住和就业。完善县域经济发展考核、激励机制，培育发展特色鲜明、发展势头好、增长后劲足的经济强县（区）。

　　三、积极深化各项改革，扩大开放与合作

推进体制改革和机制创新。深化国有企业改革，全面完成市、县所属国有企业改制。推进行政管理体制改革，加快政府职能转变，减少和规范行政审批，提高行政效率，降低行政成本，创建节约型政府。继续深化财税体制改革，推进公共财政体系建设。落实各项扶持政策，不断改善投资环境，鼓励、支持和引导非公有制经济加快发展，帮助非公有制企业不断扩大规模，提高素质，增强竞争力。大力发展开放型经济。强化招商引资工作，进一步完善激励机制，以项目为中心，创新招商方式，扩大引资领域，积极融入粤港澳，拓展闽苏浙，挺进东南亚，扩大利用外资规模。推动项目建设市场化，多渠道筹措建设资金，开工建设一批、筹划推进一批、筛选储备一批基础设施和产业项目，推进项目建设上规模、上水平。扩大对外贸易。优化出口商品结构，推进出口市场、外贸经营主体、贸易方式多元化。巩固传统出口市场，进一步开拓国际市场。引进和培育发展外向型出口创汇企业，培育外贸出口品牌，努力扩大特色优势产品出口。

　　四、加快社会事业发展，增强经济持续发展后劲

加快科技创新，加强自主创新体系建设，加快引进技术的消化吸收和再创新步伐，增强企业自主创新能力。优先发展教育事业。加快发展基础教育，巩固提高义务教育水平。深化基础教育课程改革，全面推进素质教育。稳步扩大普通高中教育规模，不断提高教育质量。积极发展学前教育、特殊教育和成人教育，开展社区教育。大力发展职业教育，建好市、县（区）职业教育中心，完成驻梧中专学校整体搬迁贺州市。发展高等教育，办好贺州学院，积极创办贺州职业技术学院。进一步完善教育投入机制，加强教育基础设施建设，改善办学条件。完善教师继续教育制度，切实提高师资水平。规范发展民办教育。大力发展文化事业。推进文艺精品创作，打造贺州文化品牌。加强文物保护和合理开发利用，开发文化产业。加快新闻出版、广播电视事业发展，全面实现20户以上已通电自然村村村通广播电视。建设市文化中心等文化设施，创建自治区级文化先进市。加强人才小高地建设，多层次培养、开发和使用各类人才。建立健全公共卫生服务体系，提高处置突发公共卫生事件、疾病预防控制和医疗救治的能力。全面改造建设乡镇卫生院，加强市直医疗卫生单位基础设施建设。扩大农村新型合作医疗覆盖面，稳步实施农村医疗救助制度。积极开展全民健身活动，加强体育设施建设，提高竞技体育水平，发展体育产业。加强民兵预备役和国防动员工作，广泛开展双拥和军民共建活动，争创全国“双拥”模范城。

　　五、建设资源节约型和环境友好型社会，实现可持续发展

加强生态建设和环境保护。实施好生态公益林保护、退耕还林、珠江防护林、石漠化治理等重点生态林业工程，加强富川西岭山自然保护区、昭平七冲原始森林自然保护区等生态功能保护区和自然保护区的保护、建设和管理。发展生态农业、生态工业和生态旅游业等生态产业。加快城镇污水处理、垃圾处理设施建设，加强重点流域、区域和重点企业的污染防治，优先保护好饮用水源，切实改善环境质量。节约利用资源，发展循环经济。加强重点行业和企业的节能降耗，严格限制高耗能、高耗材和高耗水行业发展。大力推行企业清洁生产，推进工业废物综合利用。积极推广使用散装水泥、新型建材和再生材料。高度重视水资源保护和合理利用。切实加强耕地保护和土地开发整理复垦，整顿和规范矿产资源管理秩序，节约和集约利用土地、矿产资源。加强地质灾害防治工作。全面推进计生综合改革，加快建立人口和计划生育工作新机制。坚持依法管理人口和计划生育工作，大力推行计划生育村（居）民自治。全面推进计划生育优质服务，实施农村计划生育家庭奖励扶助制度和“少生快富”扶贫工程。强化综合治理，努力降低人口性别比。加强流动人口综合治理，稳定低生育水平。

　　六、加强社会和谐基础，保障人民群众安居乐业

　　坚持以人为本，切实改善民生，加快推进和谐贺州建设。深入实施六项“民心工程”，将农村贫困残疾人危房改造、新型农村合作医疗、扶助特困复退军人等纳入民心工程的内容。完善五保村长效管理机制。抓好分批整村推进贫困村扶贫开发，帮助、支持少数民族地区加快经济发展，基本解决农村贫困人口的温饱问题。实施积极的就业政策，完善公共就业服务体系，千方百计扩大就业。建立和完善社会保障体系，扩大各类社会保险覆盖面。完善城镇居民最低生活保障制度，探索建立农村养老、医疗保险和最低生活保障制度。完善社会保险、社会救济、社会福利、优抚安置和社会互助保障体系。健全应急预案体系和社会应急机制，提高公共安全和处理突发事件的能力。深入实施药品、食品放心工程，保障人民群众的健康和安全。高度重视安全生产工作，坚决遏制重特大事故发生。深入开展“平安贺州”建设，依法严厉打击各类违法犯罪活动，强化人民调解工作，加强对群体性事件的排查、疏导和妥善处置，维护社会稳定。加强公民道德建设，继续推进桂东文明绿洲建设，广泛开展群众性精神文明创建活动，创建自治区级文明城市。加强民主法制建设。发展基层民主，全面实行政务公开、厂务公开、村务公开。加强政府法制工作，规范行政执法程序和行为，推行行政执法责任制和考评制，严格行政执法监督，提高行政执法水平。大力推广法律援助制度。扎实开展“五五”普法，推进依法治市进程。

2006年的工作任务

　　今年是实施“十一五”规划的开局之年，做好各项工作意义十分重大。今年我市经济社会发展的主要预期目标是：全市生产总值增长13%左右，财政收入增长15%左右，全社会固定资产投资增长25%左右，全社会消费品零售总额增长12%左右，外贸出口总额增长8%左右，城镇居民可支配收入增长10%左右，农民人均纯收入增长9%左右，城镇新增就业增长4%左右。

　　实现上述目标，必须按照“五业并举，工业优先，夯实基础，做大总量”的经济工作要求，突出发展园区经济、通道经济、特色经济和循环经济，着重抓好以下十个方面的工作：

　　一、大力培育发展优势产业和产业集群，壮大工业经济总量

扶持壮大优势产业和强优企业。全力推进一批新的工业项目开工建设，抓好与浙江霸力集团合作开发新路矿尾矿和大理石综合开发利用，加快英格瓷矿业公司重钙超细粉项目建设，推进桂东电子与国内上市公司──深圳特发信息公司以及灵峰药业与美国纳斯达克上市公司──美国东方生物技术有限公司的合作，支持桂东电子、金源稀土、嘉和木业等企业打造与拓展产业链。坚持扶优扶强，加大强优企业培育力度，积极引导企业通过强强联合、重组兼并等途径，做大产业规模,大力开发上下游产品，延伸产业链，培育发展优势产业集群，着重扶持桂东电力、平桂飞碟、真龙彩印等企业做大做强。同时，扶持、引导中小企业与市内外大企业配套联合发展，采取联保等多种有效方式，解决有市场、有效益的中小企业融资难、贷款难问题，促进中小企业发展。

　　加大企业技改力度。重点实施18项新开工和22项续建技改项目，完成技改投资6.5亿元，增长20%。确保桂东电子年产500万平方米中高压电子铝箔二期技改、大锰银鹤年产1.2亿只无汞碱性电池生产线、平桂飞碟年产42万吨旋窑水泥、红星纸业年产4万吨特种纸、嘉和木业年产24万立方米中纤板等技改项目竣工投产，力促合源粉体年产30万吨重钙超细粉、梅林化工年产20万吨重钙超细粉、金源稀土钕铁硼永磁材料等技改项目开工建设，加快形成一批竞争力较强的企业、品牌和产品，推动传统优势产业优化升级，提高产业竞争力。

　　大力发展园区经济。积极探索管理与经营分离的运行机制和兼顾各方利益的分配机制，加快工业园区基础设施和工业项目建设，加大园区招商引资力度，促进更多工业项目入园进区，加快承接东部产业转移基地建设步伐。旺高工业区新入区企业要达10家以上，实现销售收入3亿元以上，上缴税金3000万元以上。加快各县（区）工业小区建设，促进县域工业发展。

　　二、加强城市基础设施建设，加快推进城镇化

　　加快交通基础设施建设。继续实施交通基础设施建设大会战，加快建设洛湛铁路、桂梧高速公路、广贺高速公路贺州段等“南北贯通、五龙出海”大通道项目，桂梧高速公路八步至平乐段、国道323线莲塘至鹰阳关二级公路以及钟山至富川、钟山至恭城贺州段三级公路要建成通车。全面实施路网建设工程，加快建设昭平至蒙山黄村、八步经黄姚至昭平二级公路，开工建设9个通乡油路或水泥路工程，续建昭平至仙迴等农村公路工程。加快市客运中心、贺州港信都和昭平作业区建设。开工建设贺街、信都二级客运站，建成10个五级农村客运站和6个沿江便民码头。

　　大力推进能源建设。加快上程电站、下福电站、金牛坪电站等重点水电项目建设，积极开展桂东火电厂和上程抽水蓄能电站的前期工作。开工建设望高220千伏输变电工程。积极推进与区内外电网公司的合作，加强电力调度和供应，缓解电力供应紧张的矛盾。

　　加快城市基础设施建设。实施 “四年一大变”城市建设方案，不断完善城乡规划，完成太白湖公园、火车站站前广场及西约街、新宁街片旧城区控制性详规和城市设计以及城市消防、排水、电力专项规划。突出抓好一批改善城市景观环境的重点工程的开工和续建，推进汇豪国际城、远东国际城、香港商贸城等工程续建。加快路网建设，完成建设路、贺州大道、商业步行街改造和江北东路建设，开工建设八达西路、光明大道、公园大道、平安东路等道路延长线和新建江南新区路网项目，加速城区的外延拓展。加快龟石供水二期工程管网铺设，进一步完善城市基础设施，提升城市品位。加强城市公共交通建设。加快发展房地产业，推进商住小区开发，加快建设经济适用住房，规范房地产市场　　和物业管理。加快各县城区和重点小城镇建设，完善城镇基础设施，加快推进城镇化。

　　加强城市管理和经营。巩固“南珠杯”竞赛成果，完善城市管理长效机制，继续开展市容市貌专项治理，加强营运三轮车辆管理，搞好城市的亮化、净化、美化、绿化。加大经营城市力度，做好土地收购储备和经营，积极推进城市基础设施和公用设施经营权的有偿转让，吸引社会资本参与城市建设。

　　三、统筹城乡协调发展，积极推进社会主义新农村建设

　　推进新农村建设试点工作。按照新农村建设的总体要求，搞好总体规划，有计划、有步骤、有重点地推进新农村建设，突出抓好25个新农村建设试点工作，带动面上新农村建设。加大财政对农业和农村公共事业的投入，加强农村道路、水利、能源、教育、卫生、文化等基础设施和公共设施建设。全面落实各项支农惠农政策，大力发展劳务经济，多渠道增加农民收入。

　　加快发展品牌农业。进一步推进农业结构调整，稳定粮食生产，加快建设无公害蔬菜、脐橙、烟叶、马蹄、茶叶、梅李、松脂林、速丰林、桑蚕、食用菌等优势农产品基地，扩大规模，提高质量。推进瘦肉型猪、优质肉牛、优质家禽的规模化、标准化养殖和产业化经营，加快大水面渔业开发，建立名优鱼网箱养殖基地。做好无公害农产品、绿色食品、有机农产品的申报认证工作，努力打造一批农产品品牌。

　　提高农业企业化水平。加大以农产品加工为重点的农业龙头企业的引进、扶持发展力度，巩固提高重点龙头企业上规模、上档次，打造企业品牌，力争新增规模农业企业50家以上。加强龙头企业与农民的利益联结，加快发展订单农业。大力发展农民专业合作经济组织，力争新增农民专业合作协会20家以上。完善鲜活农产品运输“绿色通道”，搞活农产品流通。

　　大力发展生态农业。推进农产品标准化生产，深入实施无公害农产品行动计划，建设20万亩无公害标准化生产示范基地和200个标准化养殖示范场。加强农产品安全质量标准体系建设和农产品产地环境质量、农业投入品质量安全监测工作，建立健全重大动植物疫病防控长效机制。加快建设生态富民家园，大力推广“猪+沼+果+灯+鱼”等生态农业模式及其配套技术，建设沼气池1.8万座，新建生态富民示范村5个以上。

　　发展壮大县域经济。以发展非公有制经济为主，大力推进农村工业化、城镇化和农业企业化，尤其要全力抓好招商引资和县域工业，壮大新财源，促进财政增长，增强县（区）经济实力。

　　四、巩固提高商贸旅游业，提高第三产业整体水平

做大做强旅游业。启动创建中国优秀旅游城市的工作，加快景区景点和旅游基础设施建设，重点抓好贺州温泉二期工程续建，完善姑婆山、黄姚古镇等景区设施，开发农业观光园等新的景区景点，打造旅游品牌。创新旅游促销方式，加强与旅游热点城市的区域合作，积极拓展客源市场。加大特色饮食、休闲娱乐等相关产业的配套力度，开发旅游商品，延伸旅游产业链。积极举办节庆游、乡村游等活动，推动商业和旅游互动发展。加快旅游饭店宾馆星级的培育、评定，开展旅游景区质量等级评定工作。

　　大力发展商贸服务业。完成全市商业网点规划编制，优化商业网点布局。加快建设金旗市场、鼎富装饰广场、汽车农机市场等专业市场，引进、培育商贸龙头企业。组织实施“万村千乡”市场工程，发展200家乡镇和村级“农家店”加盟连锁经营，拓展农村消费市场。运用技术创新手段改造提升商贸、餐饮等传统服务业，加快发展连锁经营、物流配送、金融保险、会计咨询、信息中介、社区服务等新兴服务业。

　　五、大力推进重大项目建设，保持投资对经济增长的拉动

　　推进重大项目建设。完善重大项目工作责任制，抓好一批产业项目、基础设施和社会事业重大项目开工，集中力量推动在建项目建设，全年固定资产投资突破100亿元，力争完成120亿元。重点推进122个投资规模500万元以上的项目开工,完成投资33亿元以上；加快36个投资规模2000万元以上的续建项目建设进度,完成投资40亿元以上。加强项目建设的跟踪、监控和组织协调,确保新项目按期开工,计划竣工投产项目按质按期建成投产。

　　做好项目前期工作。规划、筛选和储备一批关系发展全局的重大项目，并做到常年筛选、动态跟踪、滚动储备，重点推进桂东火电厂、年产200万吨水泥厂、桂东林浆纸一体化项目和上程抽水蓄能电站、永州至贺州高速公路、市污水处理厂、物流中心等项目的前期工作。

多渠道筹措建设资金。完善重大项目建设的选择、滚动、责任和激励的工作机制，做好项目的论证、包装、推介和申报工作，加强与上级有关部门的衔接和沟通，积极争取国家和自治区资金、项目和政策支持，加强银企合作，提升招商引资水平，激发社会资本的活力，确保各类建设资金有新的增长。

　　六、进一步深化改革，提高对外开放水平

　　深化各项改革。深入推进以产权制度改革为核心的国有企业改革，抓紧完成市、县所属国有企业改制工作。深化国有资产管理体制改革，完善国有资产监督管理体系，加强国有资产的运营和监管。扎实推进以乡镇机构、农村义务教育和县乡财政体制改革为主要内容的农村综合配套改革，巩固农村税费改革成果。继续深化部门预算改革，推动财政国库管理制度改革，依法理顺政府采购管理体制，建立和完善财政专项资金集中管理系统，不断提高理财水平。进一步发展壮大非公有制经济。认真落实鼓励、支持和引导非公有制经济发展的各项政策，降低门槛，放宽准入领域和条件，鼓励民间资本进入经营性基础设施、农业和公益性事业领域，鼓励和支持非公有制企业采取并购、租赁、参股等形式参与国有、集体企业重组，支持符合条件的非公有制企业申办自营进出口权，促进企业做大做强。　　加快粮食流通体制改革，统筹推进其他各项改革。

　　强化招商引资工作。突出抓好“百企入桂”和利用外资工作，筹办好首届民企招商会、房地产展示暨城市基础设施项目洽谈会、第三届农展会和石材会等招商推介活动，做好第三届中国─东盟博览会的参展招商工作，加强与广东佛山、浙江衢州等地以及柳州铁路局的交流合作，提高招商引资成效。进一步完善招商引资目标责任制和激励机制，大张旗鼓地奖励招商引资有突出贡献的单位和个人。抓好各种招商推介活动签约项目的跟踪、协调和服务工作，着力提高合同履约率、资金到位率、项目开工率。

　　扩大外贸出口。积极推进出口市场、外贸经营主体、贸易方式多元化。优化出口商品结构，提高出口商品档次，扩大传统优势产品和深加工农产品出口。巩固传统出口市场，进一步开拓国际市场。加强对重点出口企业的指导和服务，用好国家扶持政策，支持企业扩大出口，确保外贸出口突破1亿美元。

　　七、加强财税金融工作，积极促进财政增收

　　加强培植税源。加快培植税收主体和骨干税源,巩固提高增值税及所得税源,发展扩大营业税源,重视中小税种税源，提高财政收入占生产总值的比重。突出抓好投资和项目建设，发挥投资对财政增长的拉动作用。加大对利税大户、重点企业的扶持力度，搞好国有资本运营,增加可用财力。建立健全财政收入增长激励机制，充分调动各方面共促财政增长的积极性。

　　强化税收征管。依法加强税源和费源管理，加强重点税源、重点企业和重点投资项目税源情况的监控和征管，提高收入征管水平，确保应收税费及时足额征收入库。

　　优化支出结构。优先保障工资正常发放、机关正常运转和突发应急支出，增加对基础教育、科技文化、公共卫生、公检法司等方面投入，足额安排好社会保障资金和救灾资金。加大对农业和农村发展的支持力度。压缩一般性财政支出，集中财力办大事。

　　增强金融对经济增长的支持。加强与金融机构的沟通，争取更多的信贷支持。鼓励和支持商业银行拓宽服务领域，增加信贷投放量。加快信用体系建设，改善金融发展环境。加快市内商业银行机构升格步伐，建立与城市发展相适应的金融体系。

　　八、加快发展社会各项事业，统筹经济社会协调发展

　　积极推进科技创新。继续推进第三轮创新计划，加快新技术、新产品的引进、开发和应用，加强企业研发中心建设，帮助支持企业开发具有自主知识产权的专利、产品和品牌，增强企业核心竞争力和产品自主创新能力。建立和完善科学技术奖励制度，健全农村科技服务体系，继续抓好科技示范基地、示范乡镇、示范村和示范户的培育和建设。

　　加强人才资源能力建设。优先发展教育事业。巩固“两基”成果，加强农村中小学建设，深入实施农村中小学危房改造工程，认真落实农村义务教育阶段学生免杂费、贫困家庭学生免费发放教材和对寄宿学生补助生活费的政策，巩固提高九年义务教育水平。积极发展学前教育和特殊教育。加快发展高中阶段教育，加快示范性高中建设步伐。全面实施振兴职业教育九大工程规划，推进市、县（区）职业教育中心建设。加快驻梧中专学校整体搬迁贺州步伐。发展高等教育，推进贺州学院建设。抓好师资培训工作，加强教师队伍建设。加强民办教育管理。加强人才培养、开发和使用。落实促进人才发展各项政策措施，以人才小高地建设为重点，推进多层次人才队伍建设，建立健全人才市场体系。

　　繁荣发展文化事业。搞好艺术精品创作，加强公共文化服务体系建设。开展“文化乡村、文化农民”创建活动，抓好基层文化馆（站）、图书馆的规范化建设，建设市文化中心等文化设施。加强网吧管理。发展文化产业，推进自治区级文化先进市的创建工作。加快发展新闻出版业，深入开展“扫黄打非”工作，维护知识产权。实施20户以上通电自然村村村通广播电视工程，扩大农村广播电视覆盖面。完成城市有线电视数字化整体转换工作。

　　加快卫生等其他事业发展。加强公共卫生体系建设，抓好42个乡镇卫生院改扩建，推进市直医疗卫生单位基础设施建设，切实加强重大疾病预防控制和妇幼保健工作，积极推进新型农村合作医疗试点和农村医疗救助。加大城乡健身场地建设投入，加快市体育中心建设，广泛开展全民健身活动，提高竞技体育水平。全面推进计生综合改革，加快建立人口和计划生育工作新机制。建立和完善以奖励扶助为主的农村计划生育利益导向机制，深入开展创建全国计划生育优质服务先进县活动，加强对流动人口计划生育管理与服务。广泛开展群众性精神文明创建活动。做好双拥优抚安置工作，继续争创全国“双拥”模范城。做好审计、统计、档案、民族宗教、外事侨务、气象、防震减灾等方面工作。关心支持妇女儿童和老龄事业，重视残疾人、工会工作。

　　大力发展循环经济。广泛开展节能降耗工作，督促引导重点行业和企业开展节能降耗。推广使用新型建材。依法强化土地管理，保护和利用好土地资源。继续整顿和规范矿产资源管理秩序，提高矿产资源综合利用水平。实施好生态林业工程，推进生态贺州建设。加强环境保护和污染治理，着重抓好重点污染企业、石材加工区污染源和贺江流域水污染治理，不断提高生态环境质量。

　　九、重视和改善民生，切实做好关系群众切身利益的工作

　　深入实施民心工程。完善五保村长效管理机制。完成550户农村贫困残疾人危房改造。完成首批整村推进贫困村扶贫开发任务，新建、改扩建村屯道路57条210公里以上，建设人畜饮水工程30处，解决2.1万贫困人口饮水困难，减少农村未解决温饱贫困人口4000人，减少低收入贫困人口8000人以上。开展农村劳动力转移培训10万人次，新增劳务输出2.5万人。强化扩大就业目标责任制，认真落实各项就业再就业扶持政策，千方百计开发就业岗位，重点帮扶下岗失业人员再就业，统筹推进大中专毕业　生、退役军人、城镇新增劳动力实现就业和农村富余劳动力转移就业。

　　加强社会保障工作。完善城镇企业职工基本养老保险制度，确保企业离退休人员基本养老金按时足额发放。完善城镇职工医疗保障体系。继续扩大各项社会保险覆盖面，加强社会保险费征缴和监管。建立健全城乡低收入困难群体社会救助制度，逐步推行农村低保和规范城市低保工作，加大城乡特困群众医疗救助力度，增加农村“五保户”供养、特困户救济经费投入。

切实维护社会稳定。加强社会治安综合治理，推进“平安贺州”建设，深入开展“三无村屯”（社区）创建活动，依法严厉打击各种犯罪活动。认真落实涉及群众利益的各项政策，坚决纠正各种损害群众合法利益的行为。重视信访和人民调解工作，做好群体性事件的排查调处和处置。深入实施食品药品放心工程，保障人民群众健康和安全。落实安全生产责任制，继续抓好安全生产专项整治，坚决遏制重特大事故发生。建立健全公共突发事件应急机制和指挥、救援体系，提高维护公共安全和社会稳定能力。

　　十、加强政府自身建设，提高经济社会管理能力

　　加强行政能力建设。加快政府职能转变，在加强经济调节、市场监管的同时，更加注重履行社会管理和公共服务职能。大力推进政企分开、政资分开、政事分开、政府与中介组织分开，减少和规范行政审批行为，改善政务环境，创新行政管理方式，推行政务公开，积极发展电子政务，提高办事效率和服务水平。建立健全科学的决策程序和体制，完善公众参与、专家论证和政府决策相结合的决策机制，提高政府决策水平。

　　全面推进依法行政。推行行政执法责任制，健全行政执法监督管理体系，确保各级行政机关及其工作人员按照法定权限和程序行使职权、履行职责。自觉接受人大的法律监督、工作监督和政协的民主监督，认真听取各民主党派、工商联、无党派人士、人民团体的意见，接受新闻舆论和社会公众监督，认真办理人大代表议案、建议和政协委员提案。广泛开展“五五”普法教育，增强全民法律意识，推进依法治市进程。

　　大力加强政风建设。强化目标管理，落实工作责任，严格督促检查，不断提高政府执行力和公信力。开展“深化改革、创新发展”大讨论活动和机关效能建设，进一步精简会议、文件，把更多的时间和精力用于深入基层、调查研究、解决问题，促进各项工作落实。严格落实党风廉政建设责任制，建立完善各种监督约束防范机制，坚决纠正部门和行业不正之风，加大违法违纪案件的查办力度。认真贯彻执行《公务员法》，加强对公务员的教育、管理和监督，着力建设高素质的公务员队伍。

各位代表，我们正站在一个新的历史起点上，新的目标令人鼓舞，新的形势催人奋进。让我们紧密地团结在以胡锦涛同志为总书记的党中央周围，高举邓小平理论和“三个代表”重要思想伟大旗帜，全面落实科学发展观，在市委的正确领导下，振奋精神，开拓进取，克难攻坚，扎实工作，为实现“十一五”规划目标，全面建设小康社会而努力奋斗!
